# Oversight and Management of Women with Psoriasis in Childbearing Age

**DOI:** 10.3390/medicina58060780

**Published:** 2022-06-09

**Authors:** Ștefana Bucur, Alexandra-Petruța Savu, Ana Maria Alexandra Stănescu, Elena-Daniela Șerban, Alin-Codruț Nicolescu, Traian Constantin, Anca Bobircă, Maria-Magdalena Constantin

**Affiliations:** 1 Department of Family Medicine, “Carol Davila” University of Medicine and Pharmacy, 050474 Bucharest, Romania; stefanabucur11@gmail.com (Ș.B.); nicolescualin66@yahoo.com (A.-C.N.); traianc29@yahoo.com (T.C.); anca.bobirca@umfcd.ro (A.B.); drmagdadinu@yahoo.com (M.-M.C.); 2 2nd Department of Dermatology, Colentina Clinical Hospital, 020125 Bucharest, Romania; elenadaniela.serbann@gmail.com; 3 Roma Medical Center for Diagnosis and Treatment, 011773 Bucharest, Romania; 4 Department of Urology, “Prof. Dr. Theodor Burghele” Hospital, 050659 Bucharest, Romania; 5 Department of Internal Medicine and Rheumatology, “Dr. I. Cantacuzino” Hospital, 073206 Bucharest, Romania

**Keywords:** psoriasis, women, pregnancy, breastfeeding, outcome, management, treatment, topical/systemic drugs, biologic agents

## Abstract

Psoriasis is a complex disease with many associated comorbidities, all of which have a negative impact on a patient’s personal, social, and sexual life. There are some unique considerations in the effects of this disease among women. The average age of diagnosis in women with psoriasis is 28 years, and this onset corresponds to the fertile life of women. There is conflicting information about the effects of psoriasis on female fertility. Some studies suggest that this condition’s associated comorbidities, personal behaviors, and reduced ovarian reserve, especially due to chronic inflammation, affect women’s fertility. Another possible reason women with psoriasis are less likely to become pregnant is that their sexual intercourse frequency decreases after the condition’s onset. The available information on the effects of pregnancy on women with psoriasis is limited. According to current evidence, most women will experience an improvement in their skin condition. Studies show that patients with moderate-to-severe psoriasis are more prone to experience pregnancy complications. The management of pregnant and lactating women with psoriasis is also difficult, as the safety profile of commonly used drugs in patients with psoriasis is not entirely known.

## 1. Introduction

**Figure 1:**
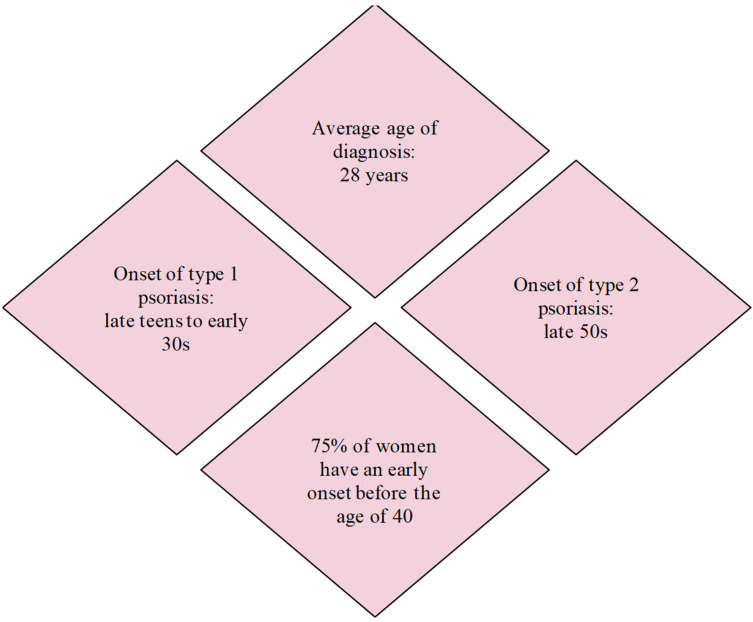
Women with psoriasis in childbearing age.

Psoriasis is a chronic, systemic inflammatory disease, with different clinical phenotypes, that affects around 2–4% of the general population [1,2]. This disease can occur at any age, with equal prevalence in both sexes. In women, psoriasis can have two peaks of onset: the first at the age of 16 to 22, and the second at the age of 55 to 60. Over 75% of women have an early onset before the age of 40 [1]. This onset corresponds to the fertile life of women, with the average age of diagnosis in women with psoriasis being 28 years (Figure 1) [3].

In addition to the cutaneous manifestations, up to 40% of patients with psoriasis have joint involvement. Psoriatic arthritis usually occurs within 5–10 years of the onset of skin disease [4].

Furthermore, psoriasis is associated with a series of comorbidities related to metabolic syndrome, including arterial hypertension, abdominal obesity, dyslipidemia, and insulin resistance [5]. Several studies have also identified a strong association between psoriasis and depression. The risk of depression among patients with psoriasis is elevated due to the social stigmatization resulting from the patient’s physical appearance [6]. Psoriasis affects a patient’s personal life, sexual intimacy, and social interactions, leading to a psychological burden [7]. Women with psoriasis are more likely to report a greater influence of the disease on their quality of life; accordingly, they are more susceptible to the development of depression [8]. An Italian study that analyzed the responses of 2391 patients at the Center for Epidemiological Studies-Depression Scale (CES-D) questionnaire observed that women were more likely than men (63 vs. 61%) to suffer from psychological symptoms [9].

For the purposes of this review, a search of the literature was performed using the PubMed and Web of Science databases. The search terms were psoriasis, women, pregnancy, oversight, and childbearing age. First, we evaluated the titles and abstracts of the articles. Articles without pertinent study objectives and articles that did not discuss the outcome of interest for this research were excluded, and relevant papers were selected. In the second stage, the full texts of the articles were read and examined in terms of inclusion and exclusion criteria.

## 2. Psoriasis and Fertility

Psoriasis is not considered to affect fertility in women or men [10]. However, some studies contradict this statement. Mathyk et al. evaluated ovarian reserve in women with psoriasis using serum levels of anti-Müllerian hormone (AMH) collected on day 3 of the menstrual cycle. Antral follicle count (AFC) and ovarian volumes were measured on the same day. AMH levels and ovarian volumes had lower values in the psoriasis group than in the control group (1.85 ± 1.13 ng/mL vs. 2.46 ± 1.21 ng/mL, p 1⁄4 0.029 and 10.43 ± 3.08 cm^3^ vs. 11.93 ± 3.01 cm^3^, p 1⁄4 0.038), but these values did not correlate with the psoriasis area severity index (PASI). The average number of antral follicles between groups was not significantly different. The PASI score did not correlate with disease duration, but serum levels of AMH, total AFC, and ovarian volume correlated inversely with disease duration [11].

Ayanoglu et al. analyzed the ovarian reserve in women with psoriasis, using estrogen, follicle-stimulating hormone (FSH), luteinizing hormone (LH), thyroid-stimulating hormone (TSH), ovarian volume, and antral follicular count (AFC), in both study and control groups. The study found that women with psoriasis had higher levels of FSH and a higher FSH/LH ratio, but lower values of AFC; these values did not correlate with the PASI score [12].

Both studies showed a reduced ovarian reserve in women with psoriasis. The causes are considered to be: autoimmunity; higher serum levels of proinflammatory cytokines (such as interleukin 23-IL-23) that may affect folliculogenesis [11]; and elevated levels of VEGF (vascular endothelial growth factor), with an increased production of keratinocytes in psoriasis leading to increased superficial cortex vascularization, which causes depletion of ovarian follicles [12].

As mentioned above, patients with psoriasis have a higher risk of metabolic and cardiovascular comorbidities that may affect the fertility of psoriatic women [13]. In addition, there are some personal behaviors, such as smoking, which have an increased prevalence among patients with psoriasis [14]. A meta-analysis that included 12 studies in determining if there is an association between smoking and infertility risk in women of reproductive age found the overall value of the odds ratio (OR) to be 1.60 [95% confidence interval (CI) 1.34–1.91] for the risk of infertility in female smokers versus non-smokers [15].

**Figure 2:**
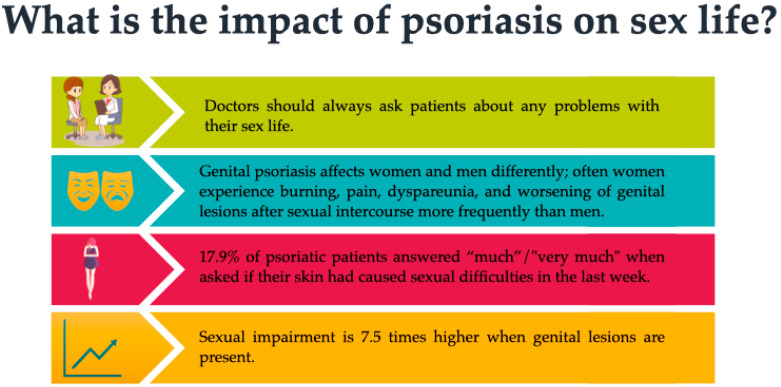
The impact of psoriasis on sex life.

Another reason for a reduced number of pregnancies among women with psoriasis could be the decreased frequency of sexual intercourse after the onset of psoriasis, especially if there is genital involvement. Meeuwis et al. found the following factors as possibly associated with decreased sexual activity (observed more in women (32.8%) than in men (19.1%)): feelings of embarrassment and reduced physical attractiveness due to psoriatic lesions, fear of partner contagiousness, the patient’s fear of transmitting psoriasis to children, decreased sexual desire on both sides, and discomfort caused by skin lesions and topical treatment [16]. Furthermore, due to genital involvement, patients have reported symptoms such as itching, stinging, burning, pain, dyspareunia, and worsening of genital lesions after sexual intercourse [17]. The possible impact of psoriasis on sex life is detailed in Figure 2.

## 3. Psoriasis and Pregnancy

Psoriasis is an autoimmune skin disease mediated by cells and molecules of the innate and adaptive immune systems. Keratinocytes are key participants in innate immunity as they recruit inflammatory dendritic cells and T cells to the skin through CCL20 chemokine. T cells are important in sustaining the activity of the disease. Myeloid dendritic cells release interleukins 23 (IL-23) and 12 (IL-12) to activate IL-17-producing T cells, T helper 1 (Th1) cells, and T helper 22 (Th22) cells to produce psoriatic cytokines, namely, interleukin 17 (IL-17), interferon-gamma (IFN-γ), tumor necrosis factor α (TNF-α), and interleukin 22 (IL-22), in psoriatic lesions. IL-17 is a key cytokine in the pathogenesis of psoriasis. These cytokines mediate the effects of keratinocytes, amplifying psoriasis inflammation [18,19].

Studies containing questionnaires and interviews with women who had experienced pregnancy while having psoriasis found that most subjects would report improvement in their cutaneous disease, some would report no change, and a small minority would report an exacerbation of their psoriasis throughout their pregnancy. Most patients are likely to experience a significant flare within four months postpartum. These changes may be due to hormonal fluctuations, especially of progesterone levels, during pregnancy and postpartum [20].

During pregnancy, estrogen and progesterone levels increase. Progesterone has been shown to stimulate the secretion of T helper 2 (Th2) cells and reduce the secretion of Th1 cytokines that maintain pregnancy [21]. Estrogen is also thought to create a shift from Th1 and Th17 to Th2 immunity with down-regulation of Th1 and Th17 cytokines and up-regulation of Th2 cytokines. Th1 responses are involved in fetal rejection as an allograft [22].

Estrogen levels in peripheral blood increase moderately from the early to late stages of pregnancy and then decrease significantly after delivery. One month postpartum, estrogen levels reach the value observed in non-pregnant women (*p* > 0.05) [23].

**Figure 3:**
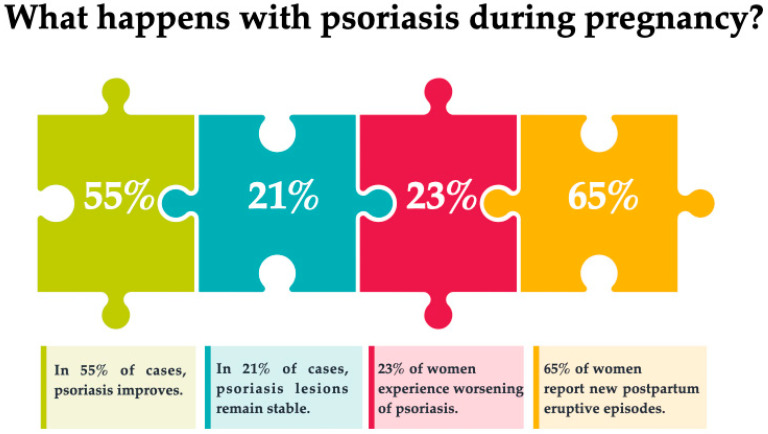
Psoriasis during pregnancy [24].

Murase et al. conducted a study that examined psoriasis body surface area (BSA) and measured hormone levels (progesterone and estrogen) in pregnancy and postpartum, comparing 47 pregnant women with psoriasis with 27 non-pregnant women with psoriasis. In total, 55% of the women experienced improvements during pregnancy, 21% had no changes, and 23% worsened. Among the postpartum women, only 9% reported improvement, 26% showed no change, and 65% worsened. Psoriasis body surface area (BSA) decreased significantly from the 10th to the 20th week of gestation (*p* < 0.001) compared to controls, while BSA increased significantly by six weeks postpartum (*p* = 0.001). In patients with psoriasis BSA of 10% or more who reported improvement, the lesions decreased 83.8% during pregnancy. There were significant or near-significant correlations between improved BSA and hormone levels: estradiol levels (*p* = 0.009, r = 0.648), estriol (*p* = 0.06, r = 0.491), and the ratio of estrogen to progesterone (*p* = 0.006, r = 0.671). There was no correlation between progesterone levels and psoriasis change [24]. Aspects of psoriasis during pregnancy are shown in Figure 3.

It has been concluded that the high estrogen levels during pregnancy are correlated with an improvement in psoriasis, instead of considering the worsened psoriatic symptoms after birth as due to lower estrogen levels. Additionally, progesterone levels did not correlate with changes in psoriasis symptoms, as had been previously suggested [20]. 

There are several studies regarding pregnancy outcomes in women with psoriasis, but with controversial results. A case–control study showed that psoriasis is an independent risk factor for cesarean delivery and is associated with recurrent abortions and chronic hypertension [25].

Another study that analyzed pregnancy outcomes in women with moderate to severe psoriasis found that women with psoriasis had a higher risk of spontaneous and induced abortions, gestational hypertension, pre-eclampsia, and premature rupture of membranes. There were no differences in cesarean deliveries, recurrent pregnancy losses, and chronic hypertension. Newborns also had higher birth weights and an increased incidence of large-for-gestational-age and macrosomia [26].

A nationwide, population-based study in Taiwan found that pregnant women with severe psoriasis had a higher risk of giving birth to low-birth-weight babies, while those with mild-to-moderate psoriasis were not at increased risk for birth complications. This may be due to increased serum and skin levels of proinflammatory cytokines that are correlated with the severity of the disease [27].

Brooms et al., in a population-based study in Sweden and Denmark, reported the following adverse pregnancy outcomes in women with psoriasis: gestational diabetes, gestational hypertension, pre-eclampsia, and emergency cesarean section. The prevalence of these consequences is higher in women with severe psoriasis and in those who have associated psoriatic arthritis. Additionally, those with severe psoriasis had a higher risk of preterm birth and low-birth-weight infants [28].

It is important to highlight the significance of psoriasis comorbidities, such as diabetes, metabolic syndrome, obesity, cardiovascular diseases, and depression, which are reported among pregnant women with psoriasis and enhance the risk of adverse birth outcomes [28,29]. Furthermore, lower use of folic acid and vitamin supplements and increased use of tobacco in the first trimester were observed in these women [29]. Smoking during pregnancy correlates with a higher risk of sudden infant death syndrome, asthma, low-birth-weight newborns, preterm birth, stillbirths, and obesity [30].

## 4. Management of Psoriasis in Pregnancy

The treatment of individuals with psoriasis consists of topical agents, phototherapy, and systemic therapies. First-line therapy for pregnant women with psoriasis consists of moisturizers and topical corticosteroids [31].

Regarding topical corticosteroids, mild-to-moderate-potency agents are preferred. High-potency topical corticosteroids should be avoided and, if necessary, should be used in small amounts for a limited time [32,33]. A topical agent that should not be used in pregnant women is tazarotene. If a woman wants to become pregnant, she should be advised to discontinue tazarotene. No human data are available regarding the excretion of these topical agents in human milk [34]. When topical therapy is insufficient due to extensive skin involvement, narrowband UVB or broadband UVB (if narrowband UVB is not available) can be used as a second-line therapy [31].

A reduction in serum folate levels has been reported after narrowband UVB but in patients with long-term phototherapy. Additionally, patients with psoriasis may have folate deficiency, regardless of their treatment [35]. During pregnancy, maternal folate deficiency has been associated with pre-eclampsia, spontaneous abortion, stillbirth, preterm delivery, low birth weight in infants, and neural tube defects in newborns [36]. Therefore, it is important for women to receive folic acid supplements during pregnancy.

Another important side effect is that narrowband UVB can exacerbate melasma, a form of hyperpigmentation on sun-exposed skin [31]. Patients require systemic therapy if the disease cannot be controlled with the therapies described above.

Systemic corticosteroids are the treatment of choice for pustular psoriasis during pregnancy (impetigo herpetiformis). Pustular psoriasis usually occurs during the third trimester but may be present earlier or in the postpartum period [37]. Regarding the side effects of systemic corticosteroids during pregnancy, there may be a slightly increased risk of cleft lip, with or without cleft palate, associated with the use of corticosteroids in the first trimester [38].

Cyclosporine, an immunosuppressive drug, may be effective in controlling severe psoriasis. It is recommended to use the minimum doses, which are less associated with side effects. Premature labor and low birth weight in newborns have been reported in cyclosporine-treated women with psoriasis [39].

The use of methotrexate and topical and oral retinoids during pregnancy is contraindicated due to their teratogenic effects. The FDA classifies methotrexate and acitretin as Category X [40]. Methotrexate, a folic acid antagonist, is associated with delayed development, craniofacial, cardiopulmonary, limb, and gastrointestinal abnormalities in newborns, even with the use of minimal doses [41]. It is also recommended that both women and men avoid conception for at least three months after taking methotrexate, although the risk of fetal abnormalities due to the use of methotrexate by men is theoretical [42].

Acitretin, a second-generation retinoid, is in the FDA’s pregnancy Category X. It should not be used to treat pregnant women with psoriasis, and it is also recommended that treatment be stopped three years before conceiving a child [40]. Treatment with acitretin during pregnancy could lead to an atypical malformation called “retinoid acid embryopathy”, which consists of cardiac, neurological, craniofacial, and thymic abnormalities [43].

Tumor necrosis factor-α (TNF-α) inhibitors are widely used to treat patients with psoriasis. They are represented by infliximab, adalimumab, etanercept, golimumab, and certolizumab-pegol [40]. Infliximab, adalimumab, and etanercept are in the FDA’s pregnancy Category B [39]. Anti-TNF-α monoclonal Ig1 antibodies, infliximab, and adalimumab cross the placenta during the third trimester and can be detected in newborns up to six months [44]. Transplacental transfer requires a neonatal Fc receptor that occurs during the second and third trimesters [45]. No congenital abnormalities or adverse pregnancy events have been reported, but the effects of exposure during children’s developmental period are still unknown [45,46,47,48]. 

One study found that treatment with anti-TNF-α agents at the time of conception may be associated with a higher risk of spontaneous abortion, but the severity of the disease and the use of other drugs may also contribute to this event [49]. Regarding the use of etanercept during pregnancy, caution should be exercised as a previous case reported infants born with VATER (V: vertebral abnormalities; A: anal abnormalities; T: tracheal problems; E: esophageal problems; R: radius or renal defects) from a patient with psoriasis and psoriatic arthritis who received etanercept during pregnancy [50]. 

Certolizumab pegol, an Fc-free pegylated anti-TNF-α agent, has the lowest placental transfer rate compared to other anti-TNF antibodies due to its structure, which consists of a humanized antigen-binding fragment (Fab’) of a monoclonal antibody conjugated to polyethylene glycol [51]. A prospective study conducted on women ≥30 weeks pregnant, with chronic inflammatory diseases in therapy with certolizumab pegol, in order to evaluate fetal exposure to certolizumab pegol during the third trimester, found that there was no to minimal placental transfer of certolizumab pegol from mothers to newborns [52]. It has also been found that there is a minimal plasma transfer of certolizumab pegol from lactating women to breast milk [53]. Regarding pregnancy outcomes, a study of a cohort of pregnant women with chronic inflammatory diseases who received certolizumab pegol did not indicate an increased risk of major congenital malformations or fetal death compared to the general population [54]. Although there is a need for further studies, certolizumab pegol appears to be a safe choice during pregnancy and the postpartum period, but the final decision on therapy is at the discretion of the treating physician, depending on the severity of the disease.

The administration of live vaccines (such as the oral polio vaccine; rotavirus vaccine; measles, mumps, and rubella (MMR) vaccine; and Bacillus Calmette–Guerin (BCG) vaccine) must be postponed until the age of 6–12 months in newborns exposed to anti-TNF agents during pregnancy [10]. There is a case report of a child exposed to infliximab during pregnancy who died of a complication of the BCG vaccine known as disseminated BCG [55].

Ustekinumab is a monoclonal antibody against the p40 subunit of IL-12 IL-23 interleukins and inhibits the actions of these cytokines, which are involved in the pathogenesis of psoriasis and other inflammatory conditions. It is classified by the FDA as Category B [40]. Data on the safety of ustekinumab during pregnancy are scarce and contradictory. There are case reports of delivery of healthy infants [56,57,58,59,60] but also of spontaneous abortions in pregnant women treated with ustekinumab [61,62].

Interleukin 17 (IL-17) inhibitors are represented by ixekizumab and secukinumab targeting IL-17A and brodalumab targeting IL-17RA [40]. Data on the use of ixekizumab and secukinumab during pregnancy are limited. Although animal studies have shown no adverse effects of ixekizumab and secukinumab on the fetus, its use during pregnancy is not recommended [63,64]. The pregnancy outcomes resulting from maternal or paternal exposure to secukinumab were analyzed using the Novartis Global Safety Database. A total of 292 such pregnancies were reported, and no safety indicators regarding spontaneous abortions or congenital malformations were identified [65]. There are no studies on the efficacy and safety of brodalumab in the treatment of pregnant or lactating women with psoriasis; therefore, the risks and benefits must be analyzed before using brodalumab as a form of treatment for this category of patients [66].

Risankizumab is a humanized immunoglobulin IgG1 monoclonal antibody specifically targeting the p19 subunit of interleukin IL-23 (IL-23A) [67]. There are limited data on the use of risankizumab in the treatment of psoriasis in pregnant women; therefore, it is recommended not to use this agent during pregnancy and to use a contraceptive method during treatment, and for a minimum of 21 weeks after its completion [68]. In a phase 2 open-label extension study that analyzed the long-term safety and efficacy of risankizumab, as used in treating patients with Crohn’s disease, three pregnancies were reported, two without any complications or abnormalities and one with fetal defects on which surgical abortion was performed [69].

Guselkumab and tildrakizumab, two other recombinant monoclonal antibodies against IL-23, are recommended to be avoided during pregnancy; the use of a contraceptive method for at least 12 weeks (guselkumab)/17 weeks (tildrakizumab) after the discontinuation of the treatment is also recommended [70].

## 5. Psoriasis and Breastfeeding

Lactating women with psoriasis may experience a post-partum flare while breastfeeding. Moreover, they are more prone to experience Koebner phenomena due to irritation from the newborn suckling. Clinically, when psoriasis affects the nipples, it will present as well-defined erythematous plaques with fine scales [71].

Psoriasis is a condition in which breastfeeding is not contraindicated, but there are some drugs used in the treatment of this disease that are contraindicated.

Regarding topical agents, tazarotene is the only contraindicated drug [72]. Topical corticosteroids may be prescribed during breastfeeding [73]. In the literature, there is a case of iatrogenic hypertension in an infant whose mother applied high-potency corticosteroids directly to the nipple [74]. Additionally, the use of topical corticosteroids on the breasts can lead to the appearance of striae, and, therefore, it is recommended that they not be applied in this region [75].

Calcineurin inhibitors, calcipotriol, and anthralin can be used in minimal doses and for a limited time, after analyzing the benefits and risks of their administration [72]. These topical medications must not be applied to the nipple and areola [72]. During pregnancy, narrowband UVB can be used safely during breastfeeding [73].

Systemic corticosteroids are reported to have a minimal transfer to breast milk, but there are no reports of adverse effects on the infants. Therefore, it is recommended to use the minimum doses for the shortest period of time. Women should also wait 4 h after ingestion of oral corticosteroids before breastfeeding [73].

Acitretin is excreted in small amounts in breast milk but, due to its potential for cumulative toxicity, should be avoided during breastfeeding [73,76]. The quantity of methotrexate transferred to breast milk is a small proportion of the maternal dose. However, it is reported that methotrexate accumulates in tissues, and due to the renal immaturity of the infant, its excretion may be affected. Therefore, it is contraindicated during breastfeeding [73,77]. The amounts of cyclosporine transferred to breast milk vary from case to case but tend to be reduced. However, it is recommended to avoid the use of cyclosporine during breastfeeding [72,73].

Regarding the use of biological agents, certolizumab pegol is excreted in small amounts in breast milk and is considered to be digested in the infant’s gastrointestinal tract. Experts recommend the use of certolizumab pegol during breastfeeding, and, in addition, some consider it to be a first-line therapy for moderate to severe psoriasis in lactating women [53,78]. Information on the use of other biologic agents during breastfeeding is limited [72].

## 6. Conclusions

Although the incidence of psoriasis in the general population is high, our understanding of pregnancy’s effects on this condition is still limited. According to current evidence, most women will experience an improvement in their skin condition. Regarding pregnancy outcomes, studies report that women with moderate-to-severe psoriasis have a higher risk of pregnancy-induced hypertensive disease, preterm birth, and low-birth-weight infants. As the safety profile during pregnancy and breastfeeding for most medications used for the treatment of individuals with psoriasis is not fully known, the final decision regarding the therapy method is at the discretion of the treating physician, depending on the severity of the disease and the associated benefits and risks.

## Data Availability

Not applicable.
